# *Eucalyptus grandis* AUX/INDOLE-3-ACETIC ACID 13 (EgrIAA13) is a novel transcriptional regulator of xylogenesis

**DOI:** 10.1007/s11103-022-01255-y

**Published:** 2022-03-16

**Authors:** Nadeeshani Karannagoda, Antanas Spokevicius, Steven Hussey, Hua Cassan-Wang, Jacqueline Grima-Pettenati, Gerd Bossinger

**Affiliations:** 1 School of Ecosystem and Forest Sciences, The University of Melbourne, Creswick, VIC 3363 Australia; 2 Centre for AgriBioscience, Agriculture Victoria, AgriBio, Bundoora, Victoria 3083 Australia; 3 Department of Biochemistry, Genetics and Microbiology, Forestry and Agricultural Biotechnology Institute (FABI), University of Pretoria, Pretoria, 0002 South Africa; 4 Laboratoire de Recherche en Sciences Végétales, Université de Toulouse III, CNRS, UPS, UMR 5546, 24 Chemin de Borde Rouge, 31320 Castanet-Tolosan, France

**Keywords:** Auxin, Aux/IAA, *Eucalyptus*, Xylogenesis, IAA13, Xylem fibre

## Abstract

**Key message:**

Our Induced Somatic Sector Analysis and protein–protein interaction experiments demonstrate that *Eucalyptus grandis* IAA13 regulates xylem fibre and vessel development, potentially via EgrIAA13 modules involving ARF2, ARF5, ARF6 and ARF19.

**Abstract:**

Auxin is a crucial phytohormone regulating multiple aspects of plant growth and differentiation, including regulation of vascular cambium activity, xylogenesis and its responsiveness towards gravitropic stress. Although the regulation of these biological processes greatly depends on auxin and regulators of the auxin signalling pathway, many of their specific functions remain unclear. Therefore, the present study aims to functionally characterise *Eucalyptus grandis AUX/INDOLE-3-ACETIC ACID 13* (*EgrIAA13*), a member of the auxin signalling pathway. In *Eucalyptus* and *Populus*, Egr*IAA13* and its orthologs are preferentially expressed in the xylogenic tissues and downregulated in tension wood. Therefore, to further investigate EgrIAA13 and its function during xylogenesis, we conducted subcellular localisation and Induced Somatic Sector Analysis experiments using overexpression and RNAi knockdown constructs of *EgrIAA13* to create transgenic tissue sectors on growing stems of *Eucalyptus* and *Populus*. Since Aux/IAAs interact with Auxin Responsive Factors (ARFs), in silico predictions of IAA13-ARF interactions were explored and experimentally validated via yeast-2-hybrid experiments. Our results demonstrate that EgrIAA13 localises to the nucleus and that downregulation of *EgrIAA13* impedes *Eucalyptus* xylem fibre and vessel development. We also observed that EgrIAA13 interacts with *Eucalyptus* ARF2, ARF5, ARF6 and ARF19A. Based on these results, we conclude that EgrIAA13 is a regulator of *Eucalyptus* xylogenesis and postulate that the observed phenotypes are likely to result from alterations in the auxin-responsive transcriptome via IAA13-ARF modules such as EgrIAA13-EgrARF5. Our results provide the first insights into the regulatory role of EgrIAA13 during xylogenesis.

**Supplementary Information:**

The online version contains supplementary material available at 10.1007/s11103-022-01255-y.

## Introduction

The phytohormone auxin regulates a variety of biological processes, including embryogenesis, root development, fruit development, tropic responses and differentiation and development of vascular tissue (Brackmann et al. 2018; Friml 2003; Smetana et al. 2019; Xu et al. 2019). In the vascular cambium, auxin plays a central role in maintaining activity, development of secondary xylem and its responsiveness to gravitational stress (Gerttula et al. 2015; Smetana et al. 2019). In the stem, auxin concentration peaks at the cambial cell division/differentiation region and gradually decreases towards the region where secondary cell wall (SCW) deposition takes place (Bhalerao and Bennett 2003; Nilsson et al. 2008), thereby regulating cambial division and differentiation, and the extent of xylem cell expansion (Brackmann et al. 2018; Mellerowicz et al. 2001; Tuominen et al. 1997). Similarly, in response to gravitropic stress, an increase in auxin can be observed in the vascular cambium of the upper (tension) side of a branch or leaning stem in angiosperm trees, demonstrating the involvement of auxin in the induction of tension wood (TW) formation (Gerttula et al. 2015). Hence, auxin flux between cambial and differentiating vascular tissues/cells is vital to ensure the active state of the vascular cambium, proper development of secondary xylem and its gravitropic responses.

Auxin flux between plant tissues, cells and cellular organelles involves different pathways and transporters. The majority of auxin produced at the shoot apex is transported to sink tissues such as the xylem via the phloem (Petrasek and Friml 2009). Directional, short distance auxin transport, for example transport of auxin from phloem to the vascular cambium and transport of auxin between xylem cells occurs via polar auxin transport, facilitated by auxin influx and efflux transporters (Goldsmith 1977; Habets and Offringa 2014; Petrasek and Friml 2009; van Berkel et al. 2013). The AUXIN TRANSPORTER PROTEIN 1 (AUX1) regulates the cellular auxin influx while PIN-FORMED1 (PIN1), P-GLYCOPROTEIN1 (PGP1) and PGP19 regulate cellular auxin efflux (Geisler et al. 2005; Krecek et al. 2009; Paciorek and Friml 2006; Ragni and Greb 2018). The nucleus subsequently perceives the auxin that enters a cell. While endoplasmic reticulum-to-nucleus flux acts as the major subcellular pathway for nuclear auxin uptake, auxin diffusion through nuclear pores contributes to a lesser degree (Middleton et al. 2018). Thus, irrespective of the developmental process, auxin signalling ultimately results in auxin perception by the nucleus.

Inside the nucleus, auxin triggers transcriptional responses. In the absence of auxin or at low auxin concentrations, INDOLE-3-ACETIC ACID INDUCIBLE (Aux/IAA) proteins bind to AUXIN RESPONSE FACTOR (ARF) protein/s, which in turn target the Auxin Responsive Elements (AuxREs) in the promoter regions of auxin-responsive target genes and recruit TOPLESS (TPL) co-repressor (Chapman and Estelle 2009; Salehin et al. 2015). This recruitment results in repression of ARFs from activating auxin-responsive target genes. When auxin enters the nucleus or at high auxin concentrations, auxin promotes the ubiquitination and subsequent 26S proteasome-mediated degradation of Aux/IAAs via the TRANSPORT INHIBITOR RESPONSE 1 (TIR1)/F-BOX PROTEINS (AFB) complex (Dharmasiri et al. 2005; Parry et al. 2009), resulting in the derepression of ARF proteins and activation of auxin-responsive target genes (Berleth et al. 2004; Wang and Estelle 2014; Woodward and Bartel 2005). However, either activation or repression of auxin-responsive target genes depends on the activating or repressing nature of the interacting ARF (Tiwari et al. 2003; Yu et al. 2014), leading to up- or downregulation of regulators of the auxin signalling pathway (Aux/IAA and ARF) and their downstream auxin-responsive target genes (Esmon et al. 2006; Schrader et al. 2004; Uggla et al. 2001, 1998). As an example, in *Populus*, PtoIAA9 binds to PtoARF5; however, in the absence of PtoIAA9, PtoARF5 can bind to AuxREs in regulatory regions and activate *HOMEODOMAIN* (*HB*) genes such as *CLASS III HOMEODOMAIN LEUCINE ZIPPER* (*HD-ZIP III*). Therefore, the PtoIAA9-PtoARF5 module regulates secondary xylem formation in *Populus* via regulating the expression of *HD-ZIPs*, which are well known to induce differentiation of cambial cells into the xylem (Xu et al. 2019). Likewise, intracellular auxin signalling in the vascular cambium or xylem eventually leads to either activation or repression of auxin-responsive target genes via Aux/IAA-ARF modules (Liu et al. 2015; Xu et al. 2019).

While auxin is well known as the principal phytohormonal regulator of cambial activity and xylogenesis, our understanding of auxin-mediated transcriptional regulation and the involvement of Aux/IAAs in these processes remain sketchy (Smetana et al. 2019; Yu et al. 2015). Aux/IAAs are composed of four domains and nuclear-localising signals. Domain I is at the N-terminus and typically contains an Ethylene Response Factor (ERF)-associated Amphiphilic Repression (EAR), which acts as a repression domain and is responsible for the recruitment of TPL co-repressor. Domain II contains the degron sequence, which regulates the turnover of Aux/IAA. Domain III and IV are collectively known as the C-terminal domain, and they are responsible for homo- and heterodimerisation with Aux/IAAs and ARFs, respectively (Luo et al. 2018). The Aux/IAA families are diverse in size, as the number of members can range from one in *Marchantia* (Bowman et al. 2017) to 100 in *Brassica* (Li et al. 2017). The Aux/IAA families in *Arabidopsis*, *Eucalyptus* and *Populus* are composed of 29, 26 and 35 members, respectively (Kalluri et al. 2007; Liscum and Reed 2002; Yu et al. 2015). Although genome-wide analyses of the *Aux/IAA* family have been conducted for about 33 plant species (Luo et al. 2018), to date only a few reports have been published on functional roles of Aux/IAAs in cambium and secondary xylem development. To the best of our knowledge, to date, only four *Aux/IAAs* (*EgrIAA4* from *Eucalyptus* and *PttIAA3*, *PtoIAA9*, and *PtrIAA14* from *Populus*) have been functionally characterised with regards to their roles in the vascular cambium in woody species (Liu et al. 2015; Nilsson et al. 2008; Xu et al. 2019; Yu et al. 2015). One such example is *PttIAA3*, which reportedly supports the maintenance of the vascular cambium and represses secondary xylem development in *Populus* by stimulating anticlinal and inhibiting periclinal cell divisions within the vascular cambium (Nilsson et al. 2008). Similarly, overexpression of a stabilised version of *PtoIAA9* in *Populus* curbs secondary xylem development by affecting xylem cell differentiation, expansion and formation of xylem vessels, via its interactions with PtoARF5 (Xu et al. 2019). In addition, *PtrIAA14.1* from *Populus* reduced the expression of *AtHB8* in transgenic *Arabidopsis* and also caused alterations in vascular patterning (Liu et al. 2015). Also, overexpression of a stabilised version of *EgrIAA4* in *Arabidopsis* leads to delayed lignification of interfascicular fibres and inhibited lignification in xylary fibres, demonstrating its involvement in auxin-mediated regulation of xylem fibre development and SCW deposition (Yu et al. 2015). Although PttIAA3, PtoIAA9, PtrIAA14 and EgrIAA4 demonstrate the significance of Aux/IAAs in auxin-mediated transcriptional regulation of cambial maintenance and xylogenesis, such regulatory roles remain poorly understood for other Aux/IAAs.

Orthologs of *Arabidopsis IAA13* in *Eucalyptus*, *Populus* and *Pinus*, are known to be differentially expressed during both wood (Li et al. 2009; Yang et al. 2014) and reaction wood formation (Chen et al. 2015; Gerttula et al. 2015; Mizrachi et al. 2015; Yu et al. 2015). This suggests their potential involvement not only in xylogenesis but also in mediating responsiveness to gravitropic stresses. Similarly, it has been observed that in *Arabidopsis AtIAA13* (AT2G33310) is preferentially expressed in xylem (Cassan-Wang et al. 2013; Yu et al. 2015). We, therefore, hypothesise that among the uncharacterised *Aux/IAAs*, *EgrIAA13* (Eucgr.H02914) is possibly a novel regulator of xylogenesis. Here we report the findings of Induced Somatic Sector Analysis (ISSA) in vivo transformation experiments. We used up- and down-regulating constructs of *EgrIAA13* to demonstrate its role in regulating the development of secondary xylem cells. We further explored protein–protein interactions of EgrIAA13 and identified IAA13-ARF modules, which we suggest might be involved in EgrIAA13-mediated transcriptional regulation of xylogenesis. Our results provide the first insights into the biological role of EgrIAA13 as a novel transcriptional regulator of secondary xylem formation.

## Materials and methods

### Plant material

For ISSA, *Eucalyptus grandis* × *camaldulensis* clones (Saltgrow) were purchased from Yuruga nursery (Queensland). *Populus alba* ‘*pyramidalis’* L. cuttings were sourced from trees growing at the University of Melbourne (Creswick Campus, Victoria) and were treated with plant cutting powder (Yates, Australia) for rapid root formation in cutting beds. All plants were maintained under controlled glasshouse conditions, where a 16 h photoperiod (by supplementary lighting when necessary) and optimum day and night temperatures (between 21–25 °C and 14–17 °C, respectively) were maintained. All plants were irrigated regularly with tap water and were fertilised with slow-release fertiliser (Osmocote, Yates, Australia) (Spokevicius et al. 2007).

### Phylogenetic and protein motif analyses

Conserved motifs in full-length amino acid sequences of Group G Aux/IAAs (EgrIAA13, EgrIAA10, PoptrIAA12, PoptrIAA11, AtIAA13, AtIAA12, AtIAA11 and AtIAA10) (Yu et al. 2015) were determined using MEME Suite 5.3.0 (http://meme-suite.org/index.html) where the distribution of motifs, maximum number of motifs, minimum motif width, and the maximum motif width parameters were set to zero or one per sequence, five, six and 50 (Bailey et al. 2009; Bailey and Elkan 1994). MUSCLE in MEGA-X was used to align these amino acid sequences, and then a Maximum Likelihood tree was constructed (using default settings) (Jones et al. 1992; Kumar et al. 2018).

### In silico analysis of expression patterns

Two in silico analyses were conducted to determine expression patterns of *EgrIAA13* in the whole plant (EucGenIE version, 1.0, https://eucgenie.org/) (Hefer et al. 2011) and expression patterns of *PoptrIAA12* (aspen ortholog of *EgrIAA13*) during different stages of xylogenesis in *Populus tremula* (using AspWood version 3.0, http://aspwood.popgenie.org/aspwood-v3.0/) (Sundell et al. 2017). Similarly, using AspWood (Sundell et al. 2017), expression patterns of the aspen orthologs of *EgrIAA3B*, *EgrIAA4*, *EgrIAA9A*, *EgrIAA11*, *EgrIAA15A*, *EgrIAA20*, *EgrIAA29*, *EgrIAA31* and *EgrIAA33A* (*Populus* best hit from BLASTP, Phytozome v12.1, https://phytozome.jgi.doe.gov/pz/portal.html) were compared with those of *EgrIAA13* as they have been previously reported to be preferentially expressed in vascular tissues (Yu et al. 2015).

### Generation of constructs for ISSA, yeast-2-hybrid and transient gene expression experiments

The synthetic, domesticated Phytobrick version of the *EgrIAA13* coding DNA sequence (CDS) (Hussey et al. 2019) was cloned into overexpression (pCAMBIA 1305.1 GW +) and RNAi knockdown (pCAMBIA 1305.1 GW-) Gateway enabled cassettes (driven by Cauliflower mosaic virus 35S promoter (35S)) using Gateway LR Clonase™ II Enzyme mix (Invitrogen) according to the manufacturer’s instructions (Spokevicius et al. 2007). The empty (without CDS) pCAMBIA 1305.1 GW + (EVC +) and empty (without CDS) pCAMBIA 1305.1 GW- (EVC-) vectors were used as controls. Subsequently, sequence-validated constructs (Macrogen, Korea) were introduced into *Agrobacterium tumefaciens* AGL1 by electroporation according to the standard protocol (Creux et al. 2013; Sambrook and Russell 2001). For the yeast-2-hybrid assay, we used in-house amplified *E. globulus* cDNA (*EgARF2B* and *EgARF6A* using Eucgr.B03551 and Eucgr.D00264 CDS specific primers, respectively) and received constructs containing *E. grandis* CDSs, *EgrARF4* (Eucgr.B02480), *EgrARF5* (Eucgr.F02090) and *EgrARF19A* (Eucgr.C03293) from LRSV, Toulouse University III / CNRS Toulouse, France and *AtIAA12* (AT1G04550), *AtIAA13* (AT2G33310), *AtARF5* (AT1G19850) and *AtARF10* (AT2G28350) from the *Arabidopsis* Biological Resource Center. According to the manufacturer’s instructions, these *Eucalyptus* and *Arabidopsis Aux/IAA* and *ARF* CDSs were gateway cloned into TaKaRA yeast-2-hybrid vectors, pGBKT7 (bait or binding domain–BD) and pGADT7 (prey or activation domain–AD), respectively. For transient gene expression experiments, full-length CDS of *EgrIAA13*, amplified using primers pairs: 5’-ATGGAAGCTCCACCTGCTC-3’ and 5’-TATCGGCTTTCTCATTTG-3’, was cloned as a C-terminal fusion in frame with the green fluorescent protein (GFP) into pK7FWG2.0 vector (Karimi et al. 2002) and expressed under the control of Cauliflower mosaic virus 35S promoter (35S) (Wang et al. 2005).

### Transient gene expression in tobacco protoplasts

Protoplasts for transfection were obtained from suspension-cultured tobacco (*Nicotiana tabacum*) BY-2 cells according to the method described in Wang et al. (2005). Typically 0.2 ml of protoplast suspension was transfected with 50 µg of either 35S:EgrIAA13:GFP or 35S::GFP (control) construct. Transfected protoplasts were incubated for 16 h at 25 °C and examined for GFP fluorescence signals using a Leica TCS SP2 laser scanning confocal microscope. Images were obtained with a 40 × water immersion objective. All transient expression assays were independently repeated three times.

### Construction of in silico protein–protein interaction networks

The in silico analysis to predict potential interacting partners of AtIAA13 in *Arabidopsis* was performed on the BioGRID database (version 4.2.191, https://thebiogrid.org/) where an interaction network was generated by selecting *Arabidopsis* as the model organism and by setting the minimum number of evidence filter to three (Chatr-Aryamontri et al. 2017; Stark et al. 2006). The in silico analysis to predict potential interacting partners of EgrIAA13 in *Eucalyptus* was performed using the STRING database (version 11.0, https://string-db.org) (Szklarczyk et al. 2017), where the amino acid sequence of EgrIAA13 was used as the query sequence, and *Eucalyptus* was selected as the model organism. This search was conducted in the confidence mode, where the required minimum interaction score was set to 0.9 (highest score). All seven active interaction sources (text mining, experiments, database, co-expression, neighbourhood, gene fusion and co-occurrence) were selected, and the remaining parameters were kept at default values (Song et al. 2019). These interaction networks and the ARF expression data from previous TW studies were analysed to identify the most suitable candidate ARFs for the protein–protein interaction experiments.

### Yeast-2-hybrid assay

Sequence-verified pGBKT7 and pGADT7 constructs were used as pairs to co-transform freshly prepared competent *Saccharomyces cerevisiae* (strain AH109) following a polyethylene glycol/Lithium acetate mediated yeast transformation protocol (Clontech). The transformed yeast was grown on several Synthetic Defined (SD) dropout media; double dropout SD medium (SD/-Trp/-Leu) which lacks tryptophan (Trp) and leucine (Leu), quadruple dropout SD medium (SD/-Trp/-Leu/-His/-Ade) which lacks Trp, Leu, histidine (His) and adenine (Ade) and quadruple dropout medium supplemented with 2 mM 3-amino-1,2,4-triazole (SD/-Trp/-Leu/-His/-Ade + 3AT). Three replicates were plated for each interaction, and plates were incubated at 30 °C for five days.

### Induced somatic sector analysis (ISSA)

Induced Somatic Sector Analysis (ISSA) protocols (including preparation of *Agrobacterium* suspension, stem inoculation, harvesting of cambial windows and GUS staining) were followed as described in Spokevicius et al. (2016), where about 30 cambial windows per construct were created in early-mid summer to generate sectors of transgenic woody tissue for subsequent harvesting (when stems have reached at least 5 mm of radial growth, usually about 11 weeks following stem inoculations), staining and analysis.

### In silico analysis of cross-reaction among *Eucalyptus IAA* genes in the RNAi knockdown experiments

The nucleotide sequences of all *Eucalyptus grandis IAAs* (Yu et al. 2015) were aligned using the Muscle alignment program in MEGA-X and the alignment was used to generate a Maximum Likelihood tree in order to identify the closest orthologs of *EgrIAA13*. Then a Pairwise Sequence Alignment (EMBOSS Water) was performed to assess the sequence similarity between *EgrIAA13* and its closest ortholog/s identified in the previous step.

### Microscopy and data analysis

**Fig. 1:**
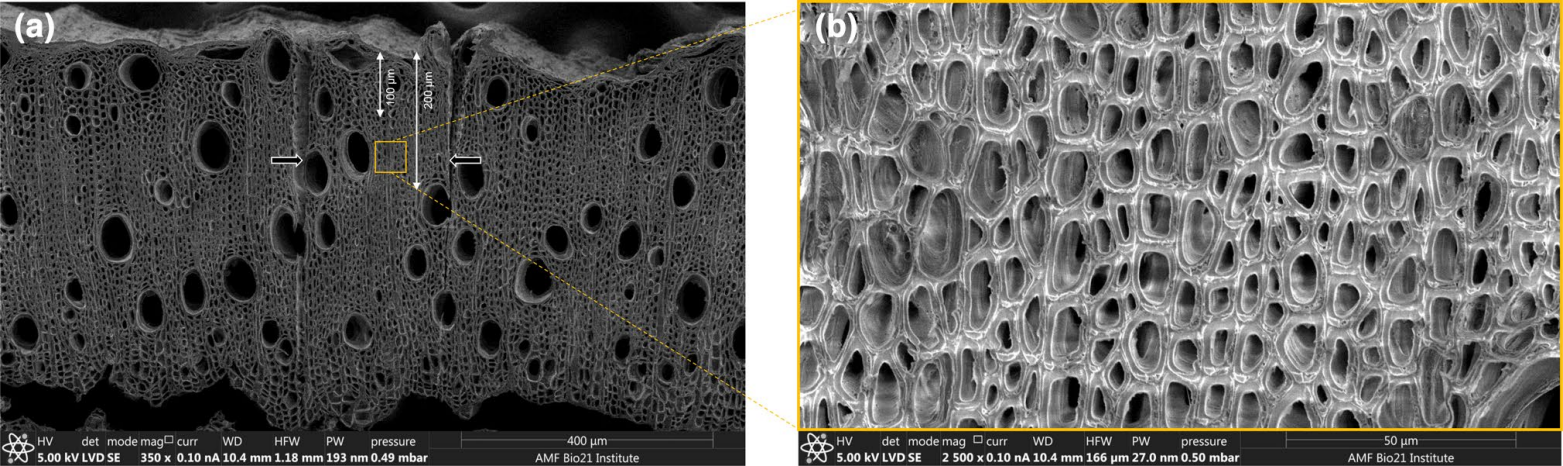
Scanning electron micrographs of transgenic sectors and cells. **a** scanning electron micrograph (350 ×) of *Eucalyptus* transverse stem section containing a transgenic sector (delineated by two radial markings on either side of the sector shown by arrows), **b** scanning electron micrograph (2500 ×) of *Eucalyptus* transgenic cells in a cross-section of the transgenic sector located between 100 and 200 μm from the cambial surface (indicated by yellow square)

Sample preparation, imaging (at 350 × and 2500 ×) and examination of secondary xylem cell ultrastructure were undertaken using scanning electron microscopy (FEI Teneo Volume Scope; low vacuum mode, acceleration voltage of either 5 kV or 10 kV) according to protocols described in Spokevicius et al. (2016). Micrographs of about 20–30 transgenic sectors and adjacent non-transgenic tissue (located between 100 and 200 μm from the cambial surface) were acquired for each construct (Fig. 1). In these micrographs, five xylem fibres from each transgenic sector and its adjacent non-transgenic tissue were arbitrarily selected (from here on collectively referred to as a ‘sector pair’), and their measurements were taken for SCW thickness (five measurements from various places on the SCW excluding the thickest corners), lumen area and cross-sectional area, using ImageJ software (https://imagej.nih.gov/ij/index.html). The SCW area was calculated as the difference between the cross-sectional area and the lumen area. For the analysis of cell counts, the region between 100 and 200 µm from the cambial surface (an area of 166 × 110 µm^2^) was selected and cell counts of five radial fibre cell lines (where available) were obtained using the same 2500 × micrographs (Fig. 1). For xylem vessel cross-sectional area, the 350 × micrographs were used, and an equal number of vessels were selected from each transgenic sector, and its adjacent non-transgenic control tissue as some micrographs/sectors contained less than five vessels. For xylem fibre length, light microscopic analysis of 20 sector pairs was undertaken. According to the protocol described in Spokevicius et al. (2016), these selected sectors were excised and macerated (Karannagoda et al. 2020). The resulting macerated fibres were mounted on a glass slide using Entellan® mounting medium (Barotto et al. 2017) and observed under an Olympus BH-2 microscope at 10 × and micrographs were acquired using a Leica DFC450 microscope camera and LAS v4 software. Five transgenic and five adjacent non-transgenic fibres were selected per sector pair from the acquired micrographs, and their lengths were measured using ImageJ software.

The selected sector pairs (Table 1) were subjected to statistical analysis by using the differences between the median values (of the fibres measured within each sector) to perform unpaired (*α* = *0.05*) t-tests as described in Spokevicius et al. (2016). In addition, these values were used to plot 95% confidence intervals, which allow to visualise and compare variance. The Inkscape 1.0 programme (http://www.inkscape.org/) was used to generate all the figures unless otherwise mentioned.

**Table 1:** Number of sector pairs used for the statistical analysis

Feature	Symbol	Plant species	Number of sector pairs (n)	Total number of cells/cell files^a^
Fibre area, lumen area, cell wall thickness and cell wall area	IAA13 +	*Eucalyptus*	21	210
	EVC +	*Eucalyptus*	27	270
	IAA13 +	*Populus*	20	200
	EVC +	*Populus*	17	170
	IAA13–	*Eucalyptus*	20	200
	EVC–	*Eucalyptus*	24	240
Vessel area	IAA13 +	*Eucalyptus*	20	90
	EVC +	*Eucalyptus*	20	84
	IAA13–	*Eucalyptus*	21	78
	EVC–	*Eucalyptus*	24	80
Fibre length	IAA13 +	*Eucalyptus*	20	200
	EVC +	*Eucalyptus*	10	100
Cell counts	IAA13 +	*Eucalyptus*	21	196^a^
	EVC +	*Eucalyptus*	15	122^a^
	IAA13–	*Eucalyptus*	25	212^a^
	EVC–	*Eucalyptus*	20	171^a^

+ and–denotes overexpression and knockdown constructs, respectively

^a^denotes the number of radial cell files that were counted for the cell count analysis. IAA13 refers to EgrIAA13.

## Results

### EgrIAA13 is a classical, nuclear localising Aux/IAA with conserved Aux/IAA domains

**Fig. 2:**
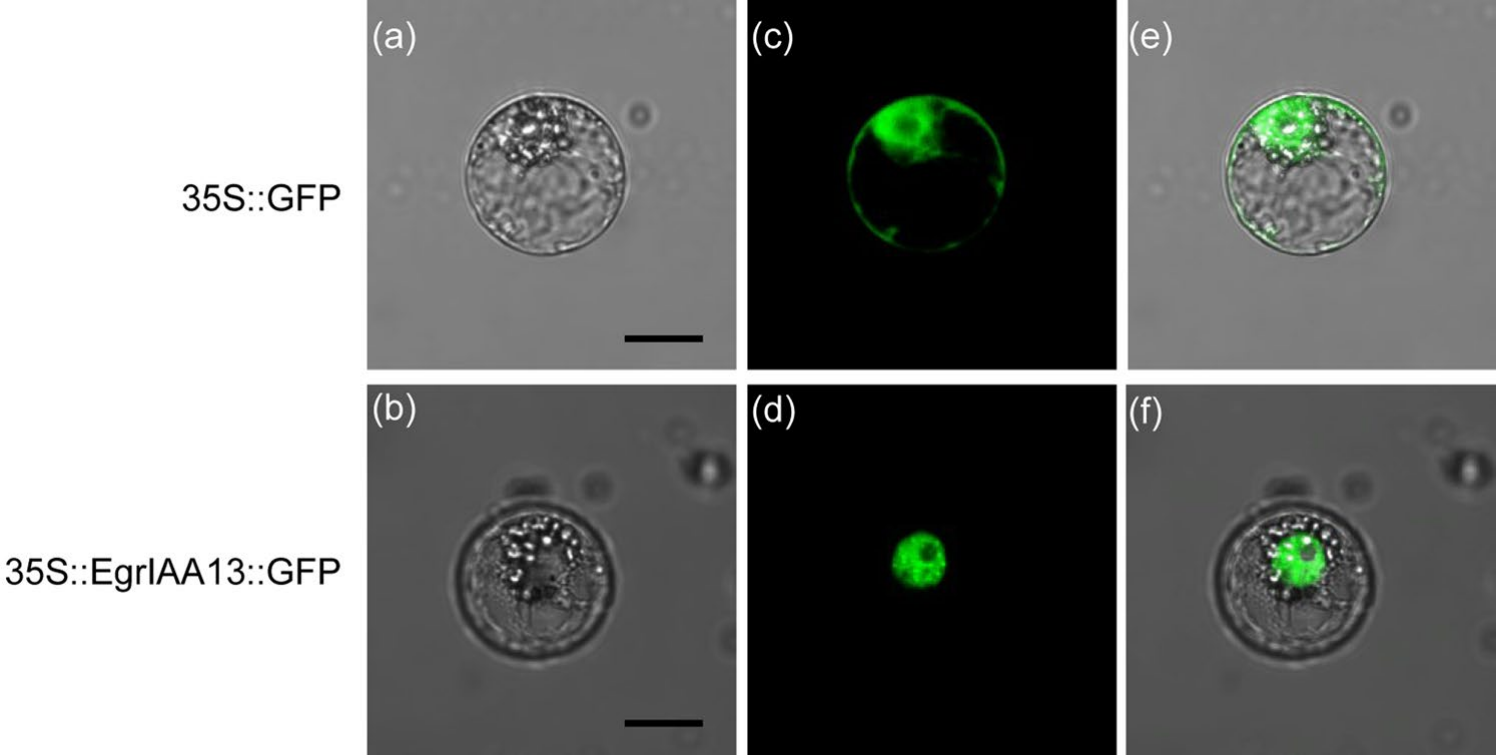
Subcellular localisation of EgrIAA13::GFP fusion protein in BY-2 tobacco protoplasts. Bright field images of **a** 35S::GFP (control GFP) and **b** 35S::EgrIAA13::GFP (EgrIAA13::GFP), green fluorescence images of **c** control GFP and **d** EgrIAA13::GFP and merged bright field and green fluorescence images of **e** control GFP and **f** EgrIAA13::GFP. Scale bars denotes 10 µm

Our phylogenetic and protein structure analyses demonstrate the close evolutionary and structural relatedness between EgrIAA13 and other members of group G Aux/IAAs (online resource 1a) and are congruent with the findings of Yu et al. (2015). The eight Aux/IAAs in group G are closely related to each other, and the closest *Populus* and *Arabidopsis* orthologs of EgrIAA13 are PoptrIAA12, AtIAA12 and AtIAA13. All group G members contain four conserved motifs (corresponding to four Aux/IAA domains) and the sequence conservation and diversity between these motifs are shown in online resource 1b. The amino acid sequence analysis demonstrates that EgrIAA13 resembles a classical Aux/IAA protein, with the four conserved domains and nuclear-localising signals (online resource 1d). Domain I contains a leucine-rich “LxLxLx” ERF-EAR motif, which can act as a repression domain by recruiting TPL co-repressor. Domain II contains the degron sequence GWPPI. EgrIAA13 also has the C-terminal domain, which is comprised of Aux/IAA domain III and IV. In addition, the transient gene expression analysis revealed that EgrIAA13 localises to the nucleus (Fig. 2) as predicted by the presence of nuclear localisation signals (online resource 1d). While the green fluorescence produced by the control GFP was observed in the nucleus, cytoplasm and along the cell membranes, that produced by the EgrIAA13::GFP fusion protein was exclusively confined to the nucleus, confirming that EgrIAA13 is capable of localising the fusion protein into the nucleus. Therefore, based on these analyses, EgrIAA13 is a *bona fide* nuclear localising Aux/IAA protein.

### *EgrIAA13* is preferentially expressed in vascular tissues

*EgrIAA13* and its orthologs are preferentially expressed in the xylogenic tissues. Our EucGenIE (Hefer et al. 2011) *in-silico* analysis and a previously published expression data set (Yu et al. 2015) demonstrated that in *Eucalyptus*, higher *EgrIAA13* expression levels were observed in the cambial, stem and root tissues, which were greater than *EgrIAA13* expression levels in other tissues including leaves, fruit capsules and floral buds (online resource 2). Among the vascular tissues, the relative expression level of *EgrIAA13* in the xylem (5.8) compared to that in the phloem (2.4) was about two-fold. In addition, the relative *EgrIAA13* expression levels vary between straight xylem (7.0) and tension xylem (2.0). The relative *EgrIAA13* expression level in the juvenile xylem was about twice as that in the mature xylem (Yu et al. 2015).

**Fig. 3:**
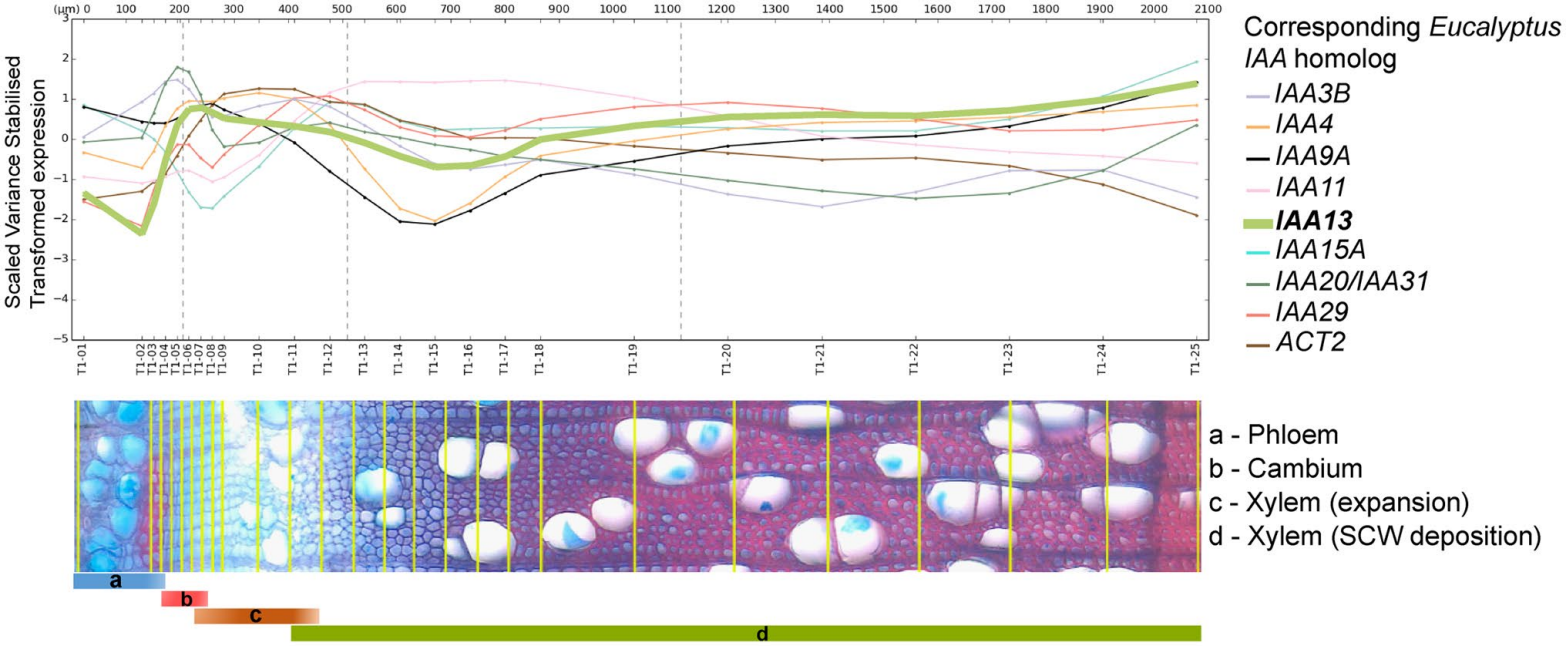
The fine-scale gene expression profiles of aspen orthologs of vascular tissue preferential *EgrIAAs*; *EgrIAA3B* (Potri.008G161100), *EgrIAA4* (Potri.005G053800), *EgrIAA9A* (Potri.002G108000), *EgrIAA11* (Potri.002G256600), *EgrIAA13* (Potri.008G172400), *EgrIAA15A* (Potri.001G177400), *EgrIAA20* (Potri.002G186400), *EgrIAA29* (Potri.006G255200), *EgrIAA20*/*IAA31* (Potri.014G111700) and reference gene *ACTIN 2* (*ACT2*) (Potri.001G309500) (Pettengill et al. 2012), during different stages of xylogenesis in *P. tremula* (revised from a figure adapted with permission from AspWood—Sundell et al. 2017)

To further dissect the preferential expression of *EgrIAA13* during vascular differentiation, the fine-scale expression of its aspen orthologs was analyzed from the AspWood database (Sundell et al. 2017). This fine-scale gene expression profile (Fig. 3) across *P. tremula* stem demonstrates a sharp rise in the transcript levels of aspen ortholog of *EgrIAA13* (*PtIAA12*/Potri.008G172400) in the cambial zone where xylem cell division and differentiation take place. Xylem cell expansion commences under this elevated transcript level; however, this transcript level gradually decreases towards the end of the xylem cell expansion zone. Although the transcript level continues to decline during the initial phase of the subsequent SCW deposition zone, then it gradually increases. This expression pattern of *PtIAA12* across the cambial (division and differentiation), xylem cell expansion and SCW deposition zones was similar to that of the aspen ortholog of *EgrIAA4* (*PtIAA8*/Potri.005G053800), which is a known regulator of fibre development and SCW formation in *Eucalyptus* (Yu et al. 2015). Although there are some similarities between the expression patterns of the aspen orthologs of *EgrIAA4*, *EgrIAA9A* and *EgrIAA13*, the expression of *PtIAA9A* (Potri.002G108000), which is the ortholog corresponding to the xylem regulator *PtoIAA9* (Xu et al. 2019), shows an increase towards the late cambial zone, a rapid decline in the xylem cell expansion zone, and then a gradual increase in maturing xylem. Based on the similarities between the expression patterns of the *EgrIAA13* ortholog and other Aux/IAA xylem regulators, as well as the downregulation of *EgrIAA13* in TW, a potential involvement of EgrIAA13 in xylogenesis is supported (Andersson-Gunneras et al. 2006; Azri et al. 2014; Chen et al. 2015; Mizrachi et al. 2015; Wang et al. 2014; Yu et al. 2015).

### EgrIAA13 alters *Eucalyptus* xylogenesis and xylem cell morphology

To confirm the potential function of *Egr*IAA13 in xylogenesis, we conducted ISSA experiments (Spokevicius et al. 2016) using overexpression (“ + ”) and knockdown (“ – ”) constructs of *EgrIAA13* to determine its effects on xylem cell morphology in both *E. grandis* × *camaldulensis* and *Populus alba* ‘*pyramidalis’* L clones. This technique involves *Agrobacterium*-mediated transformation of cambial cells *in planta*, giving rise to a transgenic wood sector which can be identified by destructive visual staining of a GUS reporter enzyme encoded by the T-DNA, and its cellular morphology compared to adjacent wild-type cells using scanning electron microscopy. An advantage of the approach is that it allows for the overall effect of a transgene (*EgrIAA13*) to be evaluated over many independent transformation events (represented by transgenic sectors) thus averaging out individual variation due to positional effects (Cooley et al. 1995; Kumpatla and Hall 1999; Twell 1992). However, this is likely to increase variation in average measurements across sectors. Also, since the precise cell file(s) that was transformed cannot be delineated by GUS staining alone, it is likely that measurements are taken of both transgenic and non-transgenic cells in a transgenic sector. Given these circumstances, we regard a *p*-value statistical threshold of 0.1 as reasonable for the present study, and we maintain that the magnitude of the effect of the transgene may be underestimated.

**Fig. 4:**
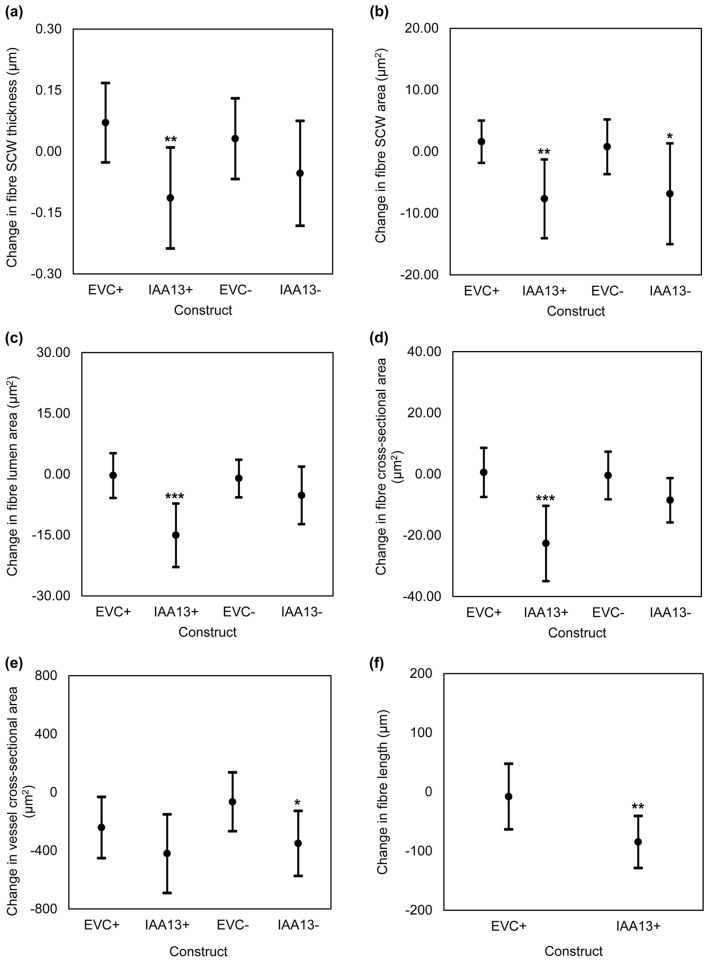
Comparison of average changes in xylem **a** fibre SCW thickness, **b** fibre SCW area, **c** fibre lumen area, **d** fibre cross-sectional area, **e** vessel cross-sectional area and **f** fibre length of *E. grandis* × *camaldulensis* xylem cells transformed with overexpression ( +) and knockdown (-) constructs of *EgrIAA13*. The number of sector pairs/cells used to make these comparisons are mentioned in Table 1. Black dots represent mean values. Error bars represent 95% confidence intervals. *** denotes *p* ≤ *0.005*, ** denotes *p* ≤ *0.05* and * denotes *p* ≤ *0.1*

**Fig. 5:**
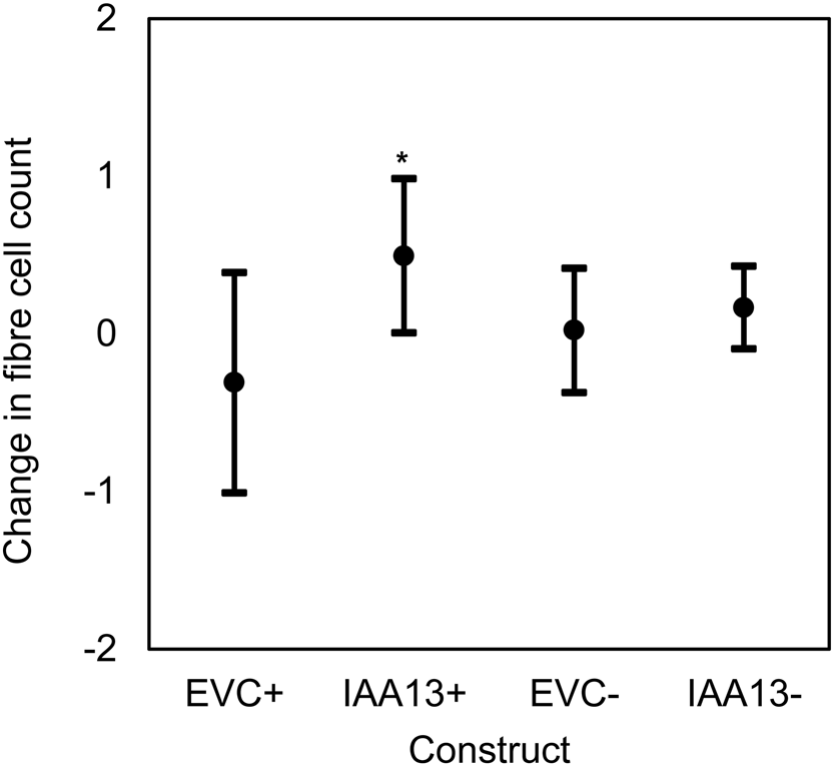
Comparison of average changes in the number of xylem fibre cells in a radial cell file of *E. grandis* × *camaldulensis* xylem transformed with overexpression ( +) and knockdown (–) constructs of *EgrIAA13*. The number of radial cell files used for these comparisons are mentioned in Table 1. Black dots represent mean values. Error bars represent 95% confidence intervals. * denotes *p* ≤ *0.1*

The *Eucalyptus* data resulting from ISSA experiments are presented in Figs. 4, 5. Relative to the EVC + (overexpression vector without the CDS), transformation with IAA13 + produced shorter and thinner xylem fibres, where average reductions were observed in xylem fibre SCW thickness of 0.18 µm (a ~ 5% decrease, *p* = *0.022*), SCW area by 9.25 µm^2^ (a ~ 10% decrease, *p* = *0.010*), lumen area by 14.72 µm^2^ (a ~ 6% decrease, *p* = *0.003*), fibre length by 76.78 µm (a ~ 5% decrease, *p* = *0.046*) and cross-sectional area by 21.99 µm^2^ (a ~ 12% decrease, *p* = *0.002*) (Fig. 4a–d and f) across 20–21 independently transformed sectors. However, no changes were observed in the cross-sectional area of the xylem vessels transformed with IAA13 + (Fig. 4e). In contrast to what was observed in *Eucalyptus*, transformation with IAA13 + did not change *Populus* xylem fibre morphology in terms of SCW thickness, SCW area, cross-sectional area, and lumen area (online resource 3). Hence, transformation with IAA13 + in this study influenced xylem fibre morphology in the homologous background *Eucalyptus* but not in the heterologous host *Populus* (data not shown).

In some instances, the high sequence homology between the transgene and the native gene might result in the silencing of both genes. This phenomenon, known as Homology Dependent Gene Silencing (HDGS), was first reported in petunia (Napoli et al. 1990; van der Krol et al. 1990) and later in various plant studies, including ISSA experiments (Baucher et al. 1996; Moller et al. 2003; Spokevicius et al. 2007; Tsai et al. 1998). However, due to the destructive sampling nature of the GUS assay, gene expression currently cannot be measured in ISSA-derived transgenic sectors. Therefore, we conducted a knockdown experiment using IAA13- in *Eucalyptus* to test whether the observed phenotypes were indeed related to overexpression or HDGS. Compared to EVC-, the xylem fibres and vessels transformed with IAA13- displayed significant average reductions in fibre SCW area of 7.62 µm^2^ (a 11% decrease, *p* = *0.094*) and vessel cross-sectional area of 284.99 µm^2^ (a 13% decrease, *p* = *0.064*), whereas fibre cross-sectional area was reduced by 8.06 µm^2^ (a 6% decrease, *p* = *0.142*) (Fig. 4b, d, e). Since our in silico analysis demonstrated that *EgrIAA13* and its only closest ortholog (*EgrIAA11*) (online resource 4a), does not share any matching 21 or 22 nucleotide sequences that can cause silencing (online resource 4b) (Elbashir et al. 2001), we conclude that it is unlikely for an untargeted *Eucalyptus IAA* to be silenced during the RNAi knockdown experiment. Therefore, we hypothesise that the observed similarity between the IAA13- and IAA13 + sectors result from HDGS.

Previously, Xu et al. (2019) reported on PtoIAA9-mediated repression during xylem cell division and expansion stages demonstrating that the overexpression of a stabilised mutant form of PtoIAA9 decreased the number of *Populus* xylem cell layers, fibre area, and vessel area. Since the smaller xylem fibre and vessel sizes observed in the present study may correspond to either a shorter radial file or an increase in the number of cells in a radial file, we hypothesised that in addition to xylem cell expansion and elongation, EgrIAA13 could also be involved in increasing the number of xylem cells in a radial file. To examine this, we first investigated if there were any obvious indentations observed in the cambial region of *Eucalyptus* IAA13 + transgenic sectors, but none were observed. Next, we examined the region between 100 and 200 µm from the cambial surface and selected an area of 166 × 110 μm (Fig. 1b) in this region and counted the number of cells in IAA13 + , IAA13-, EVC + and EVC- transgenic xylem tissue. This revealed that, on average, there were 0.8 more fibre cells (a 8% increase, *p* = *0.060*) in a radial file in IAA13 + transgenic sectors compared to EVC + ; however, there was no statistically significant difference for IAA13- (Fig. 5). Since the average amount of new xylem growth in *Eucalyptus* during the experiment was 1.5 mm, the change in the number of cells per cell file in the new xylem could be approximated to an increase of about ten fibre cells for IAA13 + compared to the EVC + . Therefore, transformation with IAA13 + increased the number of cells in a radial file.

### EgrIAA13 interacts with EgARF2, EgrARF5, EgARF6 and EgrARF19

Since several ARFs and their aspen orthologs (except the ortholog of ARF24) are expressed during xylem development (Yu et al. 2014), ARFs were prioritized for in vivo protein–protein interaction studies based on in silico interaction networks rather than expression patterns alone. The interaction network generated by the BioGRID database (online resource 5a) is based on *Arabidopsis*, and it suggests several potential interactions of AtIAA13 with other AtIAAs and AtARFs. According to this network, AtARF2, AtARF4, AtARF5, AtARF6, AtARF7, AtARF8, AtARF9, AtARF18 and AtARF19 are potentially more likely to interact with AtIAA13. Similarly, the interaction network generated by the STRING database (online resource 5b) predicts EgrIAA13 to interact with EgrARF5 and two isoforms of EgrARF19 in *Eucalyptus*. Both interaction networks highlight ARF5 and ARF19 as potential interaction partners for AtIAA13 and EgrIAA13, and therefore, EgrARF5 and EgrARF19 were selected as main candidates for in vivo protein–protein interaction studies. In addition, EgARF2, EgrARF4 and EgARF6 were also selected since their genes are differentially expressed during TW formation (similar to EgrIAA13) and because their *Arabidopsis* orthologs are known to interact with AtIAA13 (Li et al. 2011; Piya et al. 2014; Tatematsu et al. 2004; Vernoux et al. 2011; Yu et al. 2015).

**Fig. 6:**
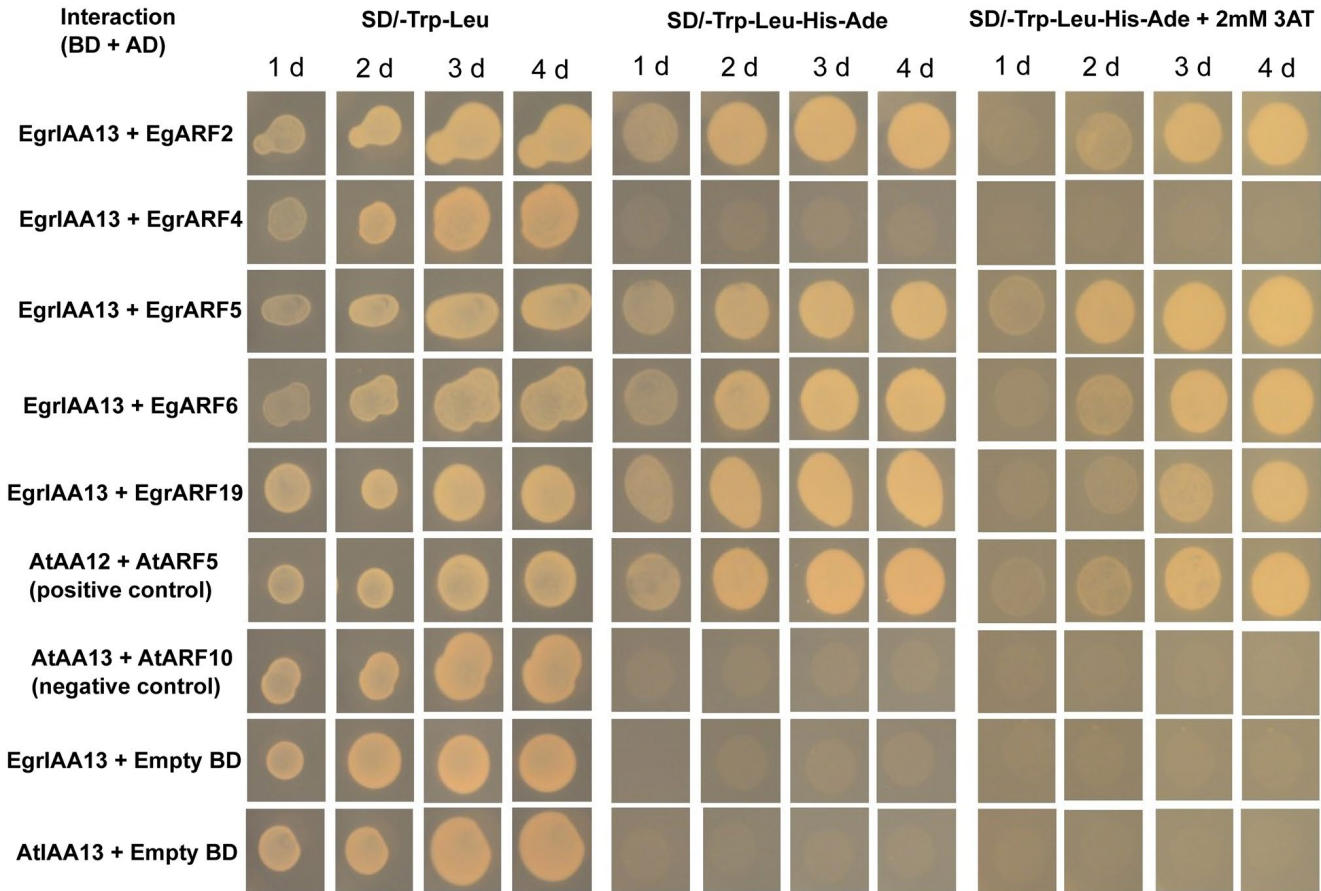
Yeast-2-hybrid assays for interactions between EgrIAA13 and selected *Eucalyptus* ARFs. The *Eucalyptus* and *Arabidopsis* IAA CDSs are cloned to pGBKT7 (BD) vectors, and the *Eucalyptus* and *Arabidopsis* ARF CDSs are cloned to pGADT7 (AD) vectors. The two combinations AtIAA13 + AtARF5 and AtIAA13 + AtARF10 were used as the positive and negative control, respectively. EgIAA13 and AtIAA13, each with empty BD vector was used to test for autoactivation. Since yeast growth did not differ between fourth and fifth days, results were shown only up to day four. As all three replicates produced similar results, the results of only one replicate are shown. The symbol ‘d’ denotes day, SD/-Trp/-Leu denotes Synthetic Defined (SD) double dropout medium (lacking tryptophan (Trp) and leucine (Leu)), SD/-Trp/-Leu/-His/-Ade denotes SD quadruple dropout medium (lacking Trp, Leu, histidine (His) and adenine (Ade)) and SD/-Trp/-Leu/-His/-Ade + 3AT denotes SD quadruple dropout medium supplemented with 2 mM 3-amino-1,2,4-triazole

Yeast-2-hybrid in vivo protein–protein interaction study was then undertaken. We plated three replicates for each interaction, and all three replicates produced similar results confirming that EgrIAA13 interacts with EgARF2, EgrARF5, EgARF6 and EgrARF19, but not with EgrARF4 (Fig. 6). To confirm that there was no self-activation of the bait construct in the absence of an interacting prey, we tested both bait constructs (*EgrIAA13* and *AtIAA13*) by co-transforming yeast with EgrIAA13 + empty AD and AtIAA13 + empty AD vector combinations and plated them on quadruple dropout medium and quadruple dropout medium supplemented with 3AT. Yeast colonies were not observed from either of these combinations, confirming that there was no self-activation. Therefore, based on our results, the EgrIAA13 modules involving EgARF2, EgrARF5, EgARF6 or EgrARF19 are potentially involved in mediating transcriptional responses of auxin-responsive xylogenesis-related genes based on their physical interaction with EgrIAA13.

## Discussion

Auxin-mediated regulation is known to control various critical stages of xylogenesis, including cell division, duration of expansion and/or the rate of expansion (Denne and Dodd 1981; Dodd and Fox 1990; Majda and Robert 2018; Mellerowicz et al. 2001; Tuominen et al. 1997). While further supporting these known aspects of auxin-mediated regulation in xylogenesis, our ISSA experiments also shed light on the role of novel Aux/IAA regulator EgrIAA13. Each result arising from our ISSA experiments (fibre SCW thickness, fibre SCW area, fibre cross-sectional area, fibre lumen area, fibre length and vessel cross-sectional area) represent the results of different cambial cell lines arising from about 20–25 independent transformation events. These results demonstrate that in *Eucalyptus*, transformation with IAA13 + produced shorter and smaller xylem fibres and transformation with IAA13- produced smaller xylem fibres and vessels, suggesting a possible involvement of EgrIAA13 in regulating xylem cell expansion. These results also show that transformation with IAA13 + increased the number of xylem cells in a radial file, indicating potential involvement of EgrIAA13 in regulating the number of *Eucalyptus* xylem cells in a radial file by increasing cell divisions. These results also revealed that fibres transformed with IAA13 + and IAA13- had similar phenotypes, suggesting IAA13 + results are most likely a consequence of downregulation through co-suppression via HDGS in a homologous genetic background. A similar observation was reported for *Eucalyptus β*-tubulin overexpression in a previous ISSA study (Spokevicius et al. 2007). However, none of the phenotypes investigated showed any changes in woody tissues of *Populus* transformed with the IAA13 + , and it is not clear from this study why this would be the case when transformed into heterologous genetic background. As previously noted, gene expression could not be confirmed in the present study due to the destructive sampling nature of the GUS assay. Based on these results, we present evidence that EgrIAA13 has a role in regulating the expansion of *Eucalyptus* xylem cells (Fig. 4d–f) and the average number of xylem cells in a radial file (Fig. 5).

The Aux/IAAs are known to mediate auxin signalling via the formation of Aux/IAA-ARF modules. Based on the results of our in vivo experiments, EgrIAA13 interacts with EgARF2, EgrARF5, EgARF6 and EgrARF19, demonstrating the possibility of forming EgrIAA13-ARF (2, 5, 6 or 19) modules. Since similar interactions have been observed between AtIAA13 and *Arabidopsis* orthologs of the above ARFs (Li et al. 2011; Piya et al. 2014; Tatematsu et al. 2004; Vernoux et al. 2011), our results also indicate possible evolutionary conservation between IAA13-ARF interactions. Furthermore, the Aux/IAA-ARF modules are known to regulate auxin-responsive genes (Hamann et al. 2002; Krogan et al. 2014; Luo et al. 2018; Xu et al. 2019; Yamauchi et al. 2019), and therefore, through heterodimerisation, EgrIAA13 is likely to prevent EgrARF2/5/6/19 from regulating downstream xylogenesis-related auxin-responsive genes.

Among these EgrIAA13-ARF modules, EgrIAA13-ARF5 and EgrIAA13-ARF19 are noteworthy. The EgrIAA13-ARF5 module is important since ARF5 is already known to play a central role in regulating cambial activity (Xu et al. 2019). For example, a recent study conducted in *Populus* demonstrated that the PtoIAA9-ARF5 module regulates *PtoHB7*/*8*, which regulates several aspects of xylogenesis, including xylem cell specification, vessel formation and xylem cell expansion (Xu et al. 2019). In *Populus*, PtoARF5 interacts with PtoIAA9 (Xu et al. 2019), while according to our results, EgrARF5 also interacts with EgrIAA13 (Fig. 6). Therefore, both EgrIAA13 and EgrIAA9 may participate in an Aux/IAA-ARF module mediated by EgrARF5. Brackmann et al. (2018) suggest that in *Arabidopsis,* AtARF5 supports xylem formation, directly activating xylem-associated genes. Therefore, EgrIAA13-ARF5 module may underlie the phenotypes observed in the present study.

**Fig. 7:**
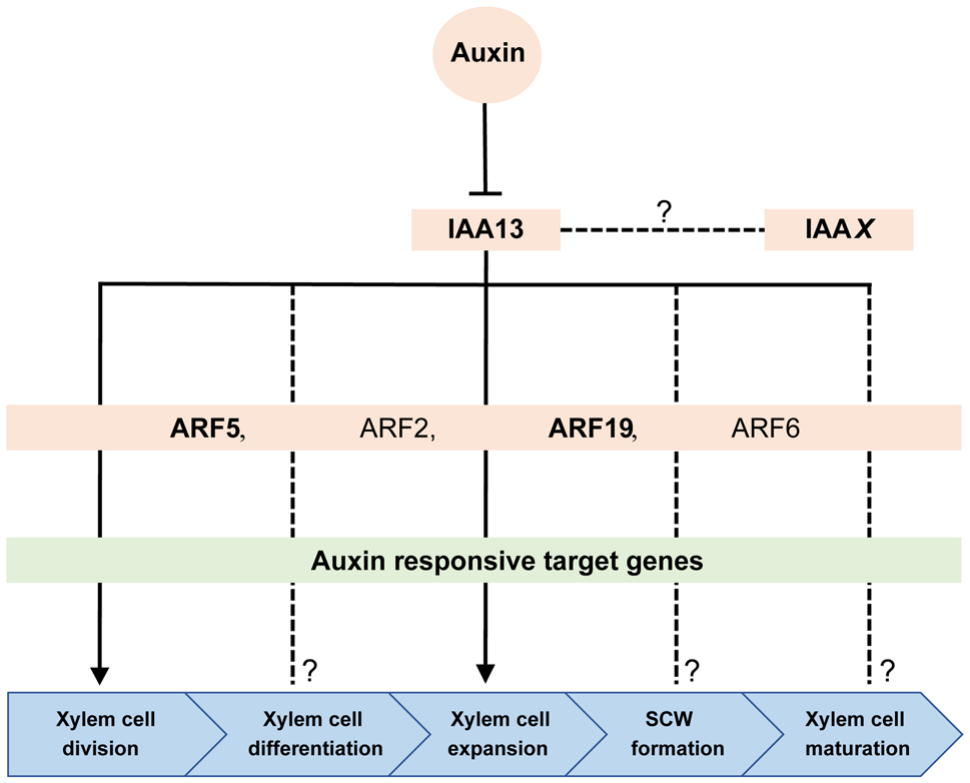
Conceptual model for EgrIAA13 dependent regulation of xylogenesis in *Eucalyptus*. IAA*X* denotes either EgrIAA3, 4, 9, 12, 15, 19 or 32. The arrows represent observed interactions/regulatory roles. Dotted lines represent unverified interactions/regulations

Unravelling the function of EgrIAA13 during xylogenesis might hold a key to expand current knowledge on the auxin-mediated transcriptional regulation of xylogenesis, which is a fundamental plant developmental process of preeminent ecological, economic and environmental importance (Andersson-Gunneras et al. 2006; Dejardin et al. 2010; FAO 2020; Groover et al. 2010; Plomion et al. 2001; Rockwood et al. 2008; Ye and Zhong 2015). Based on our study and supporting evidence from previous work, a conceptual model summarising a novel regulatory role of EgrIAA13 during xylogenesis is presented in Fig. 7. Auxin triggers proteasome-mediated degradation of Aux/IAAs (Chapman and Estelle 2009), including IAA13, which can form both homo- and heterodimers with other IAAs and ARFs, respectively. Based on our in vivo and ISSA experiments, EgrIAA13 appears to be involved in the regulation of xylem cell expansion and the number of xylem cells in a radial file (cell division), potentially via regulation of auxin-responsive target genes by EgrIAA13-ARF(2, 5, 6 and 19) module/s.

## Supplementary Information

Below is the link to the electronic supplementary material.

Supplementary file1 (DOCX 354 kb)

Supplementary file2 (PDF 1302 kb)

Supplementary file3 (PDF 302 kb)

Supplementary file4 (PDF 416 kb)

Supplementary file5 (PDF 1846 kb)

## Data Availability

The datasets generated during and/or analysed during the current study are available from the corresponding author on reasonable request.
