# Supplementary material for: Eucalyptus grandis AUX/INDOLE-3-ACETIC ACID 13 (EgrIAA13) is a novel transcriptional regulator of xylogenesis
Source: Plant Mol Biol. 2022 Mar 16;109(1-2):51–65. doi: 10.1007/s11103-022-01255-y (PMC9072461; doi:10.1007/s11103-022-01255-y)
Supplement: Supplementary file 1 — Supplementary file1 (DOCX 354 kb) [file 11103_2022_1255_MOESM1_ESM.docx]

*Eucalyptus grandis* AUX/INDOLE-3-ACETIC ACID 13 (EgrIAA13) is a novel Aux/IAA regulator of xylogenesis Plant Molecular Biology journal

Nadeeshani Karannagoda*^1,2^, Antanas Spokevicius^1^, Steven Hussey^3^, Hua Cassan-Wang^4^, Jacqueline Grima-Pettenati^4^, Gerd Bossinger^1^

^1^School of Ecosystem and Forest Sciences, The University of Melbourne, Creswick, Victoria 3363, Australia

^2^Agriculture Victoria, AgriBio, Centre for AgriBioscience, Bundoora, Victoria 3083, Australia

^3^Department of Biochemistry, Genetics and Microbiology, Forestry and Agricultural Biotechnology Institute (FABI), University of Pretoria, Pretoria, South Africa, 0002

^4^Laboratoire de Recherche en Sciences Végétales, Université de Toulouse III, CNRS, UPS, UMR 5546, 24 Chemin de Borde Rouge, 31320 Castanet-Tolosan, France

*Corresponding author: nadeeshani.karannagoda@agriculture.vic.gov.au

(

)

a

**Motif**

AtIAA13

AtIAA12

PoptrIAA12

EgIAA13

AtIAA11

AtI

AA10

EgIAA11

PoptrIAA11

100

54

99

81

65

**Domain I**

**Domain II**

**Domain III**

**Domain IV**

0

50

100

150

200

250

300

350

b

)

(

0.20

1. **Discovered Motifs Corresponding Aux/IAA Domain E-value Width of the motif**


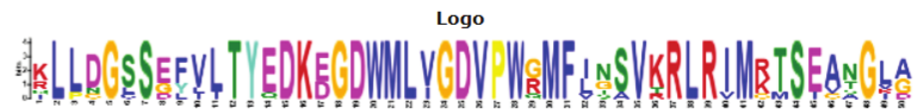
 Domain IV 8.7e-204 50


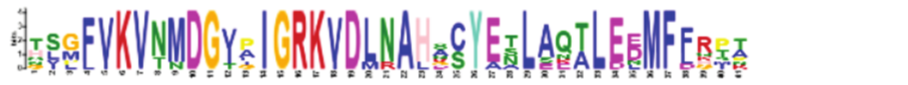
 Domain III 3.2e-149 41


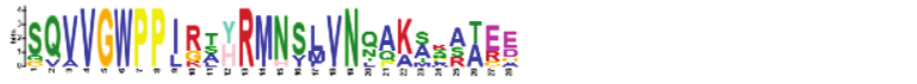
 Domain II 1.8e-088 28


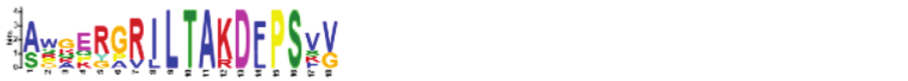
 - 6.5e-019 18


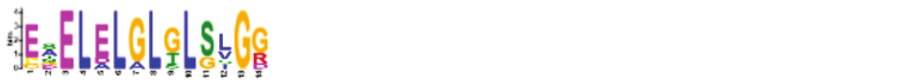
 Domain I 5.9e-018 14

1. Domain I

MEAPPARGREAAAPKRDSAGEEAE**LELGLGLS**VGGGGGGGERAGA**KR**GRILTARDFPSSVGTKRTA

Domain II

DESVSQEGGGGSPTSAS**QVVGWPPIRAYRMN**SLVNLAKAPRAEDNMSPNEKSKSKDGSEDNTRTGG

Domain III

MTDVDGREQKHI**GFVKVNMDGIPIGRKVD**LNAHACYETLAQALEDMFFRPAKTIDLTGAEENRQVKKS

Domain IV

# SKLLNGCSEFV**LTYEDKEGDWMLIGDVPWGMFLTAVKRLRIMR**TSEVNGIAPRFQQKSERQMRKPI*

Online Resource 1

Phylogenetic and sequence analysis of EgIAA13. (a) Phylogenetic analysis of closest *Arabidopsis* and *Populus* orthologs of EgIAA13 (members of Aux/IAA group G according to Yu et al. 2015). Maximum Likelihood method in MEGA-X was followed to construct the tree where the value next to the branches indicates the percentage of trees in which the associated taxa clustered together. (b) The conserved motifs in group G Aux/IAA proteins predicted using the MEME web server. The scale at the bottom indicates the relative size and the location of each domain. (c) Further information on the sequence diversity among the conserved motifs found by MEME. The E-value indicates the statistical significance of the motif. (d) The amino acid sequence of EgIAA13 demonstrating the schematic domain structure. Blue and underlined residues denote nuclear localisation signals and the region highlighted in yellow in domain II represents the degron sequence, the domains are colour coded according to those in (b).
