# Supplementary material for: Eucalyptus grandis AUX/INDOLE-3-ACETIC ACID 13 (EgrIAA13) is a novel transcriptional regulator of xylogenesis
Source: Plant Mol Biol. 2022 Mar 16;109(1-2):51–65. doi: 10.1007/s11103-022-01255-y (PMC9072461; doi:10.1007/s11103-022-01255-y)
Supplement: Supplementary file 3 — Supplementary file3 (PDF 302 kb) [file 11103_2022_1255_MOESM3_ESM.pdf]

Eucalyptus grandis AUX/INDOLE-3-ACETIC ACID 13 (EgrIAA13) is a novel transcriptional regulator of xylogenesis
Plant Molecular Biology journal

Nadeeshani Karannagoda*[1,2], Antanas Spokevicius[1], Steven Hussey[3], Hua Cassan-Wang[4], Jacqueline Grima-Pettenati[4], Gerd Bossinger[1]
[1]School of Ecosystem and Forest Sciences, The University of Melbourne, Creswick, Victoria 3363, Australia
[2]Agriculture Victoria, AgriBio, Centre for AgriBioscience, Bundoora, Victoria 3083, Australia
[3]Department of Biochemistry, Genetics and Microbiology, Forestry and Agricultural Biotechnology Institute (FABI), University of Pretoria, Pretoria, South Africa, 0002
[4]Laboratoire de Recherche en Sciences Végétales, Université de Toulouse III, CNRS, UPS, UMR 5546, 24 Chemin de Borde Rouge, 31320 Castanet-Tolosan, France

*Corresponding author: nadeeshani.karannagoda@agriculture.vic.gov.au

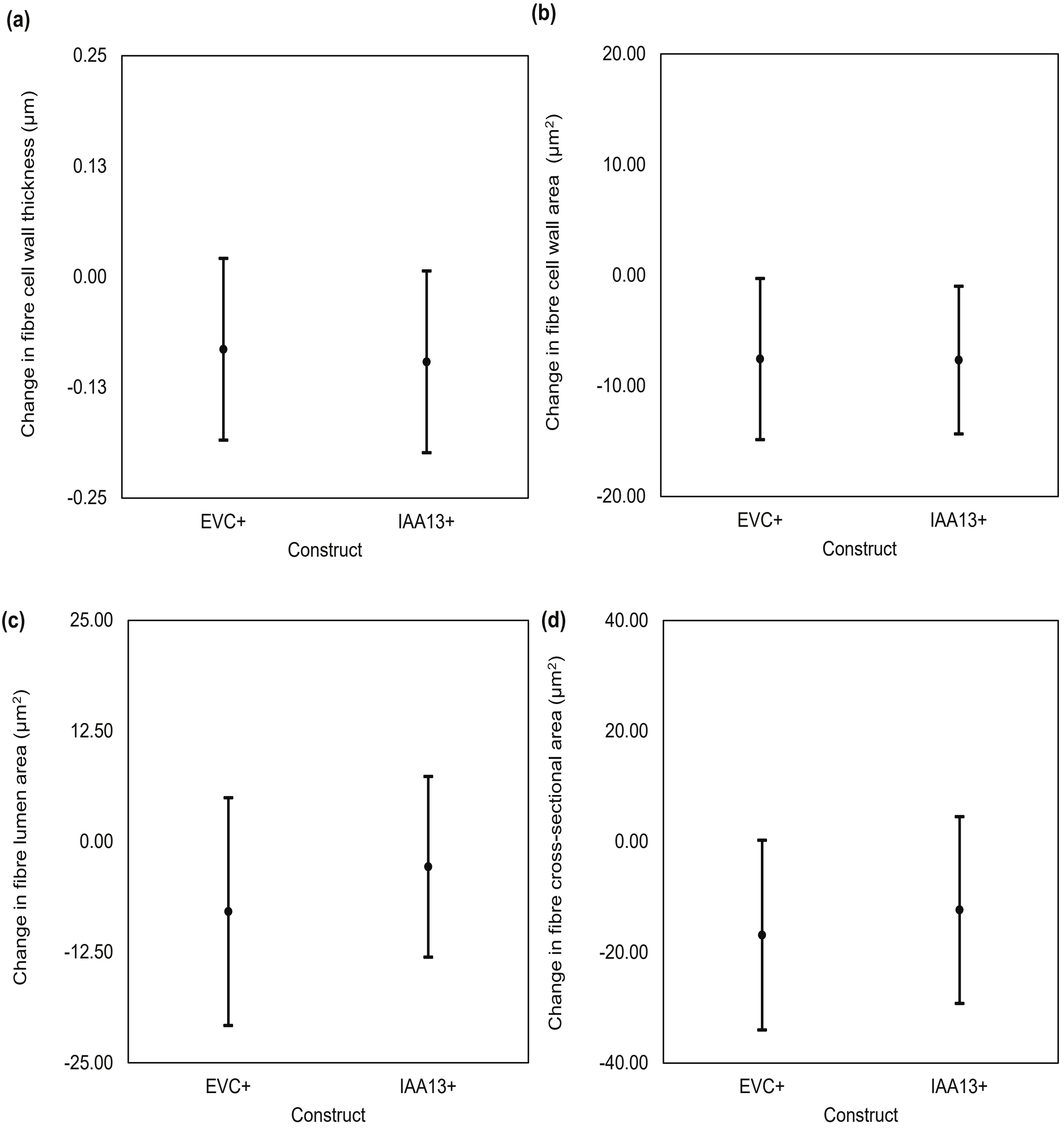

Online Resource 3
Comparison of average changes in (a) cell wall thickness, (b) cell wall area, (c) lumen area and (d) cross-sectional area of *Populus alba* xylem fibres transformed with IAA13+. The black dot represents the mean value. Error bars represent 95% confidence intervals.
