# Supplementary material for: Eucalyptus grandis AUX/INDOLE-3-ACETIC ACID 13 (EgrIAA13) is a novel transcriptional regulator of xylogenesis
Source: Plant Mol Biol. 2022 Mar 16;109(1-2):51–65. doi: 10.1007/s11103-022-01255-y (PMC9072461; doi:10.1007/s11103-022-01255-y)
Supplement: Supplementary file 4 — Supplementary file4 (PDF 416 kb) [file 11103_2022_1255_MOESM4_ESM.pdf]

Eucalyptus grandis AUX/INDOLE-3-ACETIC ACID 13 (EgrIAA13) is a novel transcriptional regulator of xylogenesis
Plant Molecular Biology journal

Nadeeshani Karannagoda*[1,2], Antanas Spokevicius[1], Steven Hussey[3], Hua Cassan-Wang[4], Jacqueline Grima-Pettenati[4], Gerd Bossinger[1]
[1]School of Ecosystem and Forest Sciences, The University of Melbourne, Creswick, Victoria 3363, Australia
[2]Agriculture Victoria, AgriBio, Centre for AgriBioscience, Bundoora, Victoria 3083, Australia
[3]Department of Biochemistry, Genetics and Microbiology, Forestry and Agricultural Biotechnology Institute (FABI), University of Pretoria, Pretoria, South Africa, 0002
[4]Laboratoire de Recherche en Sciences Végétales, Université de Toulouse III, CNRS, UPS, UMR 5546, 24 Chemin de Borde Rouge, 31320 Castanet-Tolosan, France

*Corresponding author: nadeeshani.karannagoda@agriculture.vic.gov.au

(a)

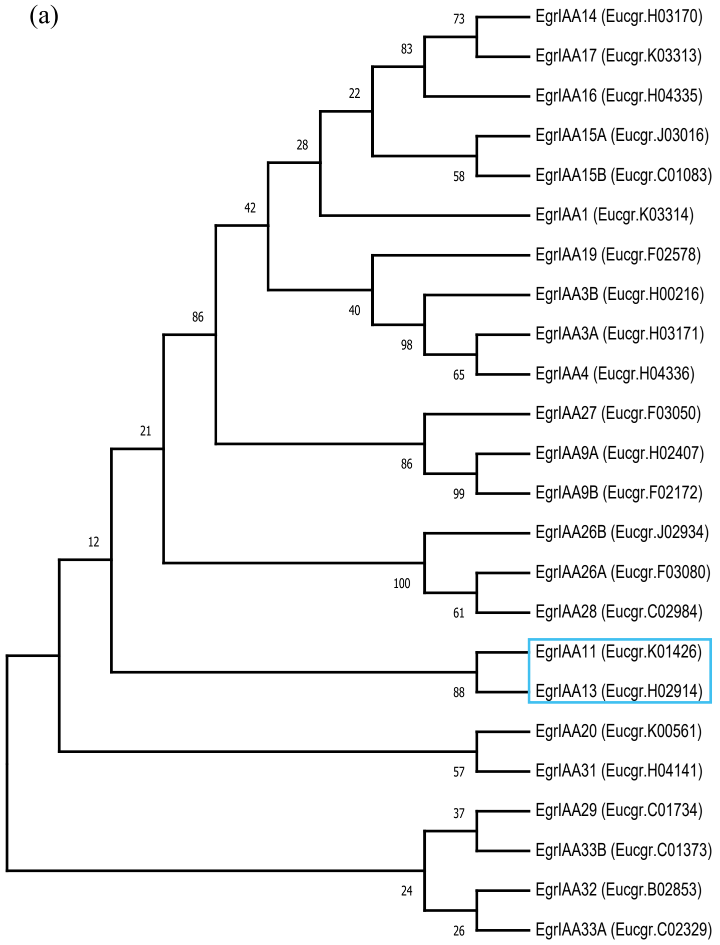

(b)

```
########################################
# Program: water
# Rundate: Sun  7 Nov 2021 01:20:45
# Commandline: water
#    -auto
#    -stdout
#    -asequence emboss_water-I20211107-012041-0067-96554010-p2m.asequence
#    -bsequence emboss_water-I20211107-012041-0067-96554010-p2m.bsequence
#    -datafile EDNAFULL
#    -gapopen 10.0
#    -gapextend 0.5
#    -aformat3 pair
#    -snucleotide1
#    -snucleotide2
# Align_format: pair
# Report_file: stdout
########################################
```

```
#=======================================
#
# Aligned_sequences: 2
# 1: EgrIAA11
# 2: EgrIAA13
# Matrix: EDNAFULL
# Gap_penalty: 10.0
# Extend_penalty: 0.5
#
# Length: 1119
# Identity:     532/1119 (47.5%)
# Similarity:   532/1119 (47.5%)
# Gaps:         430/1119 (38.4%)
# Score: 1218.5
#
#
#=======================================

EgrIAA11         117 TGCAAGTCTCTCTCTCTCTCTCTATCTCC-CTTCCTCCGCCGTCGAAGAA    165
                     ||.|||                   |||| |.|.|||
EgrIAA13           2 TGGAAG-------------------CTCCACCTGCTC-------------     19

EgrIAA11         166 ATGCAAGGAGACAGCGCCGGCGAGGCGGCGTCGCCGGCGGCGGAGCTGAC    215
                                  |||.|.|.|||||||||.|||||
EgrIAA13          20 -------------GCGGCCGTGAGGCGGCGGCGCCG--------------     42

EgrIAA11         216 CGGGTCAAAGGGGGACGACGACTACGTCGCCTCCTCGTC------GGAGG    259
                            |||.|.||                  ||||.|      |||||
EgrIAA13          43 -------AAGCGCGA------------------CTCGGCAGGAGAGGAGG     67

EgrIAA11         260 GCTCCTCGACCCCGGA---CGAGCTGGCCCTCGGGCTGACCCTCGGGGTC    306
                                 ||||   ||||||.|.|||||||||   |..||.||.|
EgrIAA13          68 ------------CGGAGCTCGAGCTCGGCCTCGGGCT---CAGCGTGGGC    102

EgrIAA11         307 GGCGACTCCCGCCCCTTCAAGTCCCCGCGGCCCGGCCC-----CGTC---    348
                     ||||.|..|.||..|..|.||    ||| |||.||.||     ||.|
EgrIAA13         103 GGCGGCGGCGGCGGCGGCGAG----CGC-GCCGGGGCCAAGCGCGGCAGA    147

EgrIAA11         349 ATCCTGACCGCCAAGGACCTGCCTTCCTTCGTCCCCGCCTCCCCCTCGCC    398
                     ||||||||.|||...|||.|.|||||||.|||               |..
EgrIAA13         148 ATCCTGACGGCCCGCGACTTCCCTTCCTCCGT---------------GGG    182

EgrIAA11         399 GCCC-------CCGCCGCCGCTGCCGGCGTCGGCGTCGGCGTCGTCGTCG    441
                     |.||       ||||||.||                         .|||
EgrIAA13         183 GACCAAGAGGACCGCCGACG-------------------------AGTC-    206

EgrIAA11         442 TCGTCTTGTTCGTCCACGCTCAGCCGGGCGGAGGGCGGCGCGGGGACTAA    491
                      |||||          |||         .||||||.||||
EgrIAA13         207 -CGTCT----------CGC---------AGGAGGGTGGCG----------    226

EgrIAA11         492 GAGGGGCGCGGATTCCGTGGCCGCTCCTACTGCTTCCAGTCAGGTAGTGG    541
                            |.||||          |.||.|||.||.|.||||||||.||||
EgrIAA13         227 -------GTGGAT----------CCCCCACTTCTGCAAGTCAGGTTGTGG    259

EgrIAA11         542 GATGGCCTCCCCTCAAAACTCACAGAATGAACGTCTTTATTAACACTC-C    590
                     |||||||.||..|.|...|..||||.||||||..|.|..|.||| ||| |
EgrIAA13         260 GATGGCCACCTATAAGGGCATACAGGATGAACAGCCTGGTCAAC-CTCGC    308

EgrIAA11         591 AAAATCTACTTC-AACT--GGAGAGTTTGACCCCGTAGTTGAGAAAAATG    637
                     |||..| ||.|| |.||  |||.|...||.|.|||           ||||
EgrIAA13         309 AAAGGC-ACCTCGAGCTGAGGACAACATGTCGCCG-----------AATG    346

EgrIAA11         638 ---ATAGCAAGCTT-----GCTGTGTTAGAGAAGATCAATA---------    670
                        |.||||||  |     |.|| |||.| ||.|| |||||
EgrIAA13         347 AAAAGAGCAAG--TCAAAGGATG-GTTCG-GAGGA-CAATACGCGTACTG    391
```

```
EgrIAA11          671 ATGCCAGAAATGA----------------CAAAAGCAGTAACTCCATGAA    704
                      .||.||..|.|||                ||||||||
EgrIAA13          392 GTGGCATGACTGATGTTGACGGCAGAGAGCAAAAGCA-------------    428

EgrIAA11          705 GGTATCGTGCCCCAAAACTTCTCCTTTTGTAAAGGTCAATATGGATGGAG    754
                        |||||.|                |||||.|||||.||.||||||||..
EgrIAA13          429 --TATCGGG----------------TTTGTGAAGGTGAACATGGATGGGA    460

EgrIAA11          755 TTGCAATTGGAAGGAAGGTAGATCTAAATGCACATCAATGCTATGAGAGT    804
                      |..|.||||||||||.||.||..|.|||||.|||...||.|||||||.|
EgrIAA13          461 TCCCCATTGGAAGGAAAGTGGACTTGAATGCTCATGCTTGTTATGAGACT    510

EgrIAA11          805 TTAGCAGAGACATTGGAGGATATGTTTGATCATCCCA-CTACAAAAGTCA    853
                      .||||..|..|.|||||.||.||||    ||.|||.| ||.|.||
EgrIAA13          511 CTAGCTCAAGCTTTGGAAGACATGT----TCTTCCGACCTGCCAA-----    551

EgrIAA11          854 ACGCACCACGTTCAAATAGGTTGATGTATCATGG----------ATTGGT    893
                                   ||.||  |||||.||.|..||          ||.||.
EgrIAA13          552 -------------AACTA--TTGATTTAACCGGGGCTGAAGAAAATAGGC    586

EgrIAA11          894 ACGAGCAACAAGTGCCTC-AAAATTGT---TGGATGGTTCATCTG-GTTA    938
                      | |..|||.|||| |||| ||..||.|   ||||||    .|||| |||
EgrIAA13          587 A-GGTCAAGAAGT-CCTCGAAGCTTCTCAATGGATG----TTCTGAGTT-    629

EgrIAA11          939 TGTTCTTACTTATGAAGACAAAGATGGAGATTGGATGCTTATTGGAGATG    988
                      .||.||.|||||||||||.|||||.|||||.||||||||.||||||||||
EgrIAA13          630 CGTGCTAACTTATGAAGATAAAGAGGGAGACTGGATGCTCATTGGAGATG    679

EgrIAA11          989 TTCCTTGGAGGTATGTAGCCCGGTG--GT------ACT----TTCATTTT   1026
                      |||||||| ||.||||..|.|..||  ||      |||    .||||...
EgrIAA13          680 TTCCTTGG-GGGATGTTCCTCACTGCCGTCAAGAGACTAAGAATCATGAG    728

EgrIAA11         1027 GATTGTTTATATTGTGTGTTACCTTATGCAAGTCCTGCACCAATTCATTT   1076
                      ||                    |||.|| ||||
EgrIAA13          729 GA--------------------CTTCTG-AAGT-----------------    740

EgrIAA11         1077 TGGCATTTGGCATTTTTC--ATATT------TAAGCTTTGAAGC-----C   1113
                        |.|  |||.||...||  |.|||      .|||      |||     .
EgrIAA13          741 --GAA--TGGAATAGCTCCAAGATTCCAACAAAAG------AGCGAGAGG    780

EgrIAA11         1114 CAAGTG-GAA-----ATAT   1126
                      |||.|| |||     ||||
EgrIAA13          781 CAAATGAGAAAGCCGATAT    799


#---------------------------------------
#---------------------------------------
```

Online resource 4

In silico analysis demonstrating the unlikelihood of silencing an untargetted *Eucalyptus IAA* during the RNAi knockdown experiments (a) Maximum likelihood tree demonstrating *EgrIAA11* as the closest ortholog of *EgrIAA13* and (b) Pairwise Sequence Alignment (EMBOSS Water) demonstrating the sequence similarity between *EgrIAA13* and *EgrIAA11* (https://www.ebi.ac.uk/Tools/services/rest/emboss_water/result/emboss_water-I20211107-012041-0067-96554010-p2m/aln).
