# Supplementary material for: Eucalyptus grandis AUX/INDOLE-3-ACETIC ACID 13 (EgrIAA13) is a novel transcriptional regulator of xylogenesis
Source: Plant Mol Biol. 2022 Mar 16;109(1-2):51–65. doi: 10.1007/s11103-022-01255-y (PMC9072461; doi:10.1007/s11103-022-01255-y)
Supplement: Supplementary file 5 — Supplementary file5 (PDF 1846 kb) [file 11103_2022_1255_MOESM5_ESM.pdf]

*Eucalyptus grandis* AUX/INDOLE-3-ACETIC ACID 13 (EgrIAA13) is a novel transcriptional regulator of xylogenesis
Plant Molecular Biology journal

Nadeeshani Karannagoda*[1,2], Antanas Spokevicius[1], Steven Hussey[3], Hua Cassan-Wang[4], Jacqueline Grima-Pettenati[4], Gerd Bossinger[1]
[1]School of Ecosystem and Forest Sciences, The University of Melbourne, Creswick, Victoria 3363, Australia
[2]Agriculture Victoria, AgriBio, Centre for AgriBioscience, Bundoora, Victoria 3083, Australia
[3]Department of Biochemistry, Genetics and Microbiology, Forestry and Agricultural Biotechnology Institute (FABI), University of Pretoria, Pretoria, South Africa, 0002
[4]Laboratoire de Recherche en Sciences Végétales, Université de Toulouse III, CNRS, UPS, UMR 5546, 24 Chemin de Borde Rouge, 31320 Castanet-Tolosan, France

*Corresponding author: nadeeshani.karannagoda@agriculture.vic.gov.au

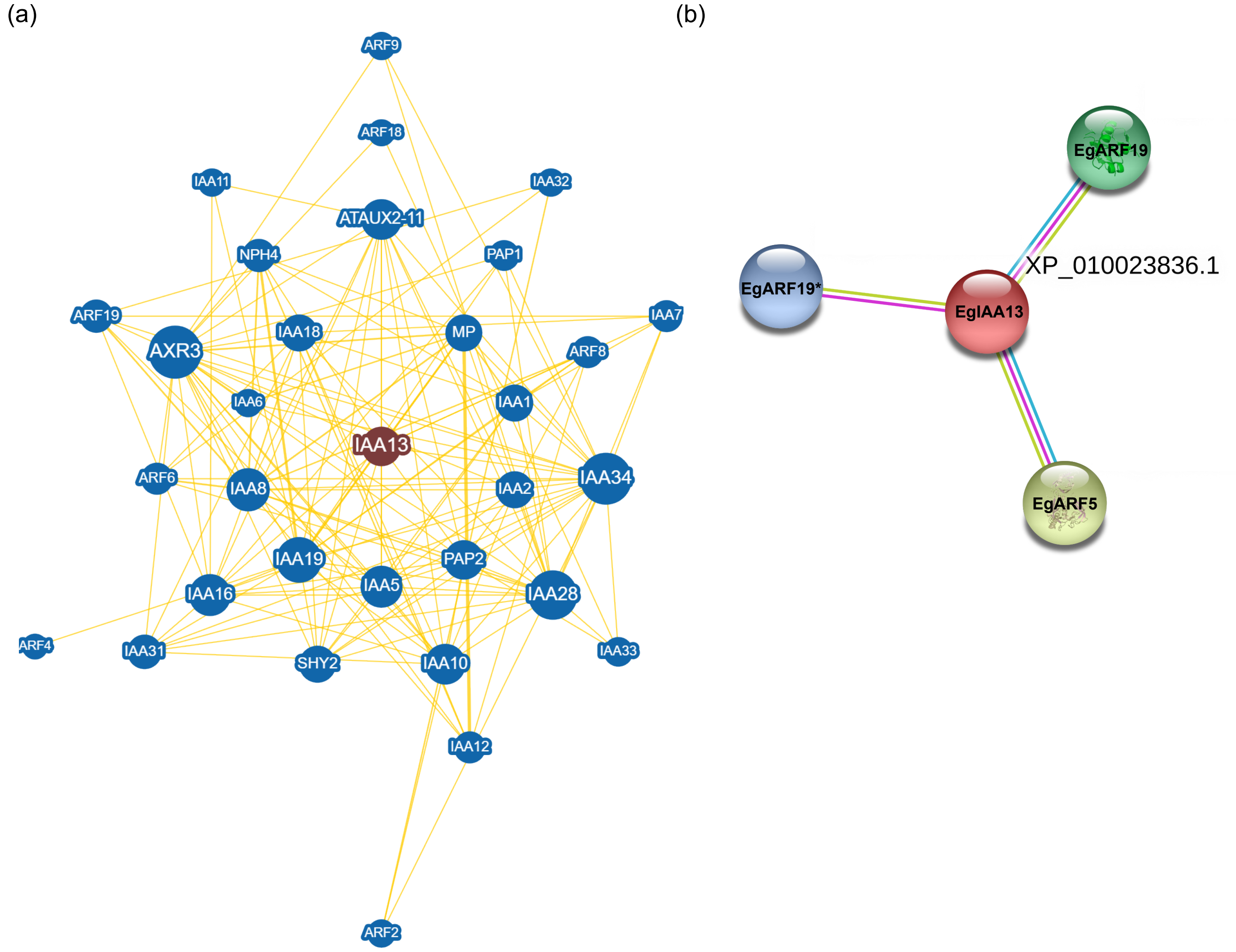


Online Resource 5

Protein-protein interaction networks predicted for (a) AtIAA13 by BioGRID database and (b) for EgIAA13 (revised from figure produced by STRING database). MP stands for MONOPTEROS (ARF5), NPH4 stands for NON-PHOTOTROPIC HYPOCOTYL (AtARF7), PAP stands for PHYTOCHROME-ASSOCIATED PROTEIN, SHY2 stands for SHORT HYPOCOTYL 2 (IAA3), AXR3 stands for AUXIN RESISTANT3 (AtIAA17) and ATAUX2-11 stands for AUXIN INDUCIBLE 2-11 (IAA4). The protein accessions corresponding to EgARF5, EgARF19 and EgARF19* are XP_010061491.1, XP_010048533.1 and XP_010049370.1, respectively. In (b) the edges connecting the nodes are colour coded; pink denotes predictions based on experimental evidence, blue denotes predictions based on database evidence and green denotes predictions based on neighbourhood evidence.
